# Serum kisspeptin and proopiomelanocortin in cystic fibrosis: a single study

**DOI:** 10.1038/s41598-022-21851-8

**Published:** 2022-10-21

**Authors:** Sabina Galiniak, Rafał Podgórski, Marta Rachel, Artur Mazur

**Affiliations:** Institute of Medical Sciences, Medical College, Rzeszów University, Rzeszow, Poland

**Keywords:** Predictive markers, Endocrine system and metabolic diseases

## Abstract

The determination of hormonal biomarkers is of increasing interest in many diseases, including cystic fibrosis (CF). Hormones that have not been estimated and described so far in CF include kisspeptin (KISS) and proopiomelanocortin (POMC), which are involved in the regulation of many processes, including appetite and fertility. Therefore, the aim of our study was to estimate the level of KISS and POMC in sera from CF patients and to determine the correlation between these hormones and clinical parameters. For this purpose, we estimated the levels of KISS and POMC in 38 CF patients and 16 healthy participants with enzyme-linked immunosorbent assay. We found significantly reduced levels of KISS and POMC in people with CF compared to healthy subjects (1.76 ± 0.46 vs. 2.27 ± 0.56 ng/mL, p < 0.05 and 6.25 ± 4.36 vs. 14.74 ± 6.24 ng/mL, p < 0.001, respectively). Furthermore, the level of both hormones was negatively correlated with age. The hormones studied did not correlate with the results of spirometry and each other. Thus, decreased KISS and POMC levels may be associated with lower body weight and delayed puberty in patients with CF.

## Introduction

Biomarkers are a growing scientific interest in modern medicine. The use of biomarkers has great potential for accurate disease diagnosis, prediction of complications, and prognosis in patients with multiple diseases who exhibit varying degrees of inflammation, infection, and accompanying organ dysfunction or failure^1–4^. Such diseases include cystic fibrosis (CF), which is the most common inherited genetically determined disease caused by a mutation in the gene for the CF transmembrane conductance regulator protein. Pulmonary failure is the main cause of death in this population, while heavy involvement of the gastrointestinal system creates significant nutritional deficiencies^5–7^. Today, due to new treatments, as well as increased awareness of the disease, patients with CF live longer so that more than half of those living with CF are adults^8^. Consequently, sexual and reproductive health is increasingly important for people with CF, as many consider parenting. Most men and some women with cystic fibrosis have reduced fertility, which is multifactorial for both sexes^9,10^ Furthermore, despite new therapeutic strategies, many nutritional disorders are still common in CF^11^. Therefore, much research has focused on hormones in CF to provide novel insights into the body’s energy homeostasis and fertility. Dysregulation of hormones that regulate appetite is common in CF and primarily affects leptin and ghrelin^12–15^. Kisspeptins (KISS) are a number of structurally related amidated peptides, which are derived from the differential proteolytic processing of a common precursor of 145 amino acids encoded by the *KISS1* gene. KISS is produced in central nervous system by neurons that can be found in discrete nuclei in the hypothalamus where regulates the GNRH secretion^16^. It is also an adipokine, however, the contribution of adipose KISS to circulating KISS is currently unknown^17^. Moreover, the roles adipose KISS plays in energy metabolism, reproduction, and homeostasis are currently unclear.

As is the case for adipokines in general, data from association studies suggested that the dysregulation of KISS during different diseases may be relevant in the pathogenesis of various diseases such as obesity, diabetes, and sepsis^18,19^. Proopiomelanocortin (POMC) is a precursor peptide that is the basis for various molecules that give rise to several biologically active peptides that are expressed primarily in the pituitary and brain. Among these are adrenocorticotropic hormone, α-, β- and γ-melanocyte-stimulating hormones (MSH), β-lipotrophin, and endorphins. POMC neurons in the central nervous system are highly heterogeneous in their regulation and action. Therefore, POMC-derived peptides can have opposing effects on appetite regulation, while α-MSH suppressing -MSH and β-endorphin, promoting appetite^20,21^. The purpose of our study was to estimate the concentration of KISS and POMC in sera of patients with CF and to determine the correlation between these hormones and clinical parameters of patients. To our knowledge, this is the first report to describe the serum concentration of KISS and POMC in CF.

## Methods

### Ethical issues

The research protocol was approved by the Bioethics Committee of Rzeszów University (2022/023). All procedures performed in studies involving human participants were in accordance with the ethical standards of the institutional and/or national research committee and with the Declaration of Helsinki of 1964 and its subsequent amendments or comparable ethical standards. The informed consent was obtained from all participants or if participants are under 16, from a parent and/or legal guardian.

### Study group

A single-center cross-sectional study was conducted in 38 CF patients aged 10 to 39 and 16 control patients. Participants were recruited from the local CF care center at the Provincial Hospital No. 2 in Rzeszow from February to October 2021.

The study included patients with a CF with confirmed diagnosis based on the determination of sweat chloride, genetics, and immune-reactive trypsin tests in neonatal age (patients born in or after 2009). The following criteria were met to enroll patients in the study: forced expiratory volume in the first second (FEV_1_) greater than 35% of predicted stable pulmonary disease, as defined by both clinical impressions and no hospitalizations within 30 days of screening. The exclusion criteria were also: CF-related diabetes, glucose intolerance, heart failure and liver failure, psychiatric disorders, lung transplantation and treatment with corticosteroids. Furthermore, patients were excluded if they could not perform spirometry and refused to participate in the study. All patients with CF have pancreatic insufficiency and received pancreatic enzyme replacement therapy (Creon 25000, Solvay Pharmaceutical Inc., Marietta, Georgia, USA). Patients were also treated with human DNase I recombinant (Pulmozyme, Genentech Inc., San Francisco, California, USA; one 2.5 mg ampoule inhaled once a day using a nebulizer), fat-soluble vitamins in the form of ADEK tablets (Scandipharm, Birmingham, Alabama, USA), supplemental nutrition drinks (Nutrison Protein Plus, Nutricia, Poland) and inhalation of 3–7% sodium chloride 3–4 times daily. Additionally, a fertility interview was conducted in adult CF patients.

Healthy patients aged 10–38 years were recruited simultaneously from the local clinic. The control group consisted of volunteers who had no medical history or physical examination of the disease. Healthy participants did not take any drugs 30 days before the study. All participants in the control group had normal pulmonary function tests. All participants had anthropometric measurements. BMI was calculated as kg/m^2^.

### Spirometry

All participants performed spirometry using a standard spirometry device (Lungtest 1000, MES, Kraków, Poland) according to recommendations^22^. We calculated the mean value of the last half year for FEV_1_ expressed as a percentage of the predicted value for age and sex.

### Blood sampling

Blood samples were collected between 8 and 10 a.m. after a night of fasting and placed in blood collection tubes. The collected serum was incubated at room temperature for at least 30 min and centrifuged (1500 × *g*, 10 min, 4 °C). Subsequently, the serum was transferred to cryovials and placed immediately in the freezer at − 80 °C until further analysis. The serum sample was not stored for more than 3 months and was thawed only once on the day of analysis.

### Blood counts and serum analysis

Blood morphology was performed using hematology analyzer (Siemens Healthineers, Germany). C-reactive protein (CRP) was estimated using the dry chemistry immunological method on a VITROS 250 analyzer (Ortho Clinical Diagnostics, Johnson and Johnson, USA).

### Kisspeptin (KISS) and pro-opiomelanocortin (POMC)

The serum concentrations of KISS and POMC after an overnight fast were measured in duplicate with previous dilution using a commercially available enzyme-linked immunosorbent assays (Wuhan Fine Biotech Co., Ltd., Wuhan, China), according to the manufacturer’s instructions and expressed as ng/mL. The limit of detection for KISS and POMC was 0.156 ng/mL, and the within-assay and between-assay coefficient of variations were lower than 8% and lower than 10%, respectively.

### Statistical analysis

All statistical analyses were performed with the STATISTICA software package (version 13.3, StatSoft Inc. 2017, Tulsa, OK, USA). Data are expressed as mean and SD, as well as range. The normality of the distribution was validated using the Shapiro–Wilk test, as well as skewness values. The Mann–Whitney *U* test and, for multiple comparisons, the Kruskal–Wallis ANOVA were used. The correlation analysis was performed using the Spearman correlation test, assuming linear dependence with α = 0.05.

## Results

Thirty-eight people with CF were recruited into the study, including 17 women (44.7%). At the same time, 16 healthy people, including 10 women (62.5%), were included in the study. Baseline characteristics, clinical laboratory values, and indices of lung function for patients with CF and healthy controls are presented in Table 1.

**Table 1:** Baseline demographic and clinical data of the study participants.

		CF	Healthy controls	p value
Sex (F/M)		17/21	10/6	
Age (years)	mean ± SD	19.58 ± 7.9	19.25 ± 7.3	0.855
	range	10‒39	10‒38	
Height (cm)	mean ± SD	157.56 ± 18.1	160.56 ± 15.1	0.598
	range	124‒188.5	130‒180	
Weight (kg)	mean ± SD	50.03 ± 13	58.75 ± 13.9	0.038
	range	22.1‒76	34‒82	
BMI (kg/m^2^)	mean ± SD	19.89 ± 2.8	22.47 ± 2.5	0.003
	range	14.4‒25.9	18.7‒25.6	
**Genotype**				
Homozygous ΔF508, n (%)		30 (78.9)	*‒*	‒
Heterozygous ΔF508, n (%)		8 (21.1)	*‒*	‒
**Clinical laboratory markers**				
WBC (10^3^/µL)	mean ± SD	9.95 ± 3.6	7.46 ± 2.3	0.022
	range	5.1‒19.3	4.3‒10.5	
NEU (%)	mean ± SD	61.01 ± 15.3	59.12 ± 6.1	0.605
	range	25.1‒82.3	50.6‒68.6	
CRP (mg/L)	mean ± SD	5.32 ± 4.8	1.92 ± 1.2	0.001
	range	0.5‒22	0.3‒4.2	
**Pulmonary function**				
FEV_1_ (% predicted)	mean ± SD	86.35 ± 27	102.4 ± 8.2	0.006
	range	35‒142	97‒127	

Data are presented as mean ± SD; differences between means were analysed using Mann–Whitney *U* test.

*BMI* body mass index, *WBC* white blood cells, *NEU* neutrophils, *CRP* C-reactive protein, *FEV*_1_ forced expiratory volume in 1 s.

There were no age and height differences between patients with CF and healthy controls. Weight and BMI were significantly lower in participants with CF. Of the CF patients, 30 were homozygous and 8 heterozygous for ΔF508. White cell levels were significantly higher in CF subjects compared to controls. The CRP was significantly higher in the CF group compared to healthy individuals. Pulmonary function indices were significantly reduced among CF participants (p < 0.01). None of the adult patients with CF had children. In the medical history, adult patients reported problems with infertility.

**Figure 1:**
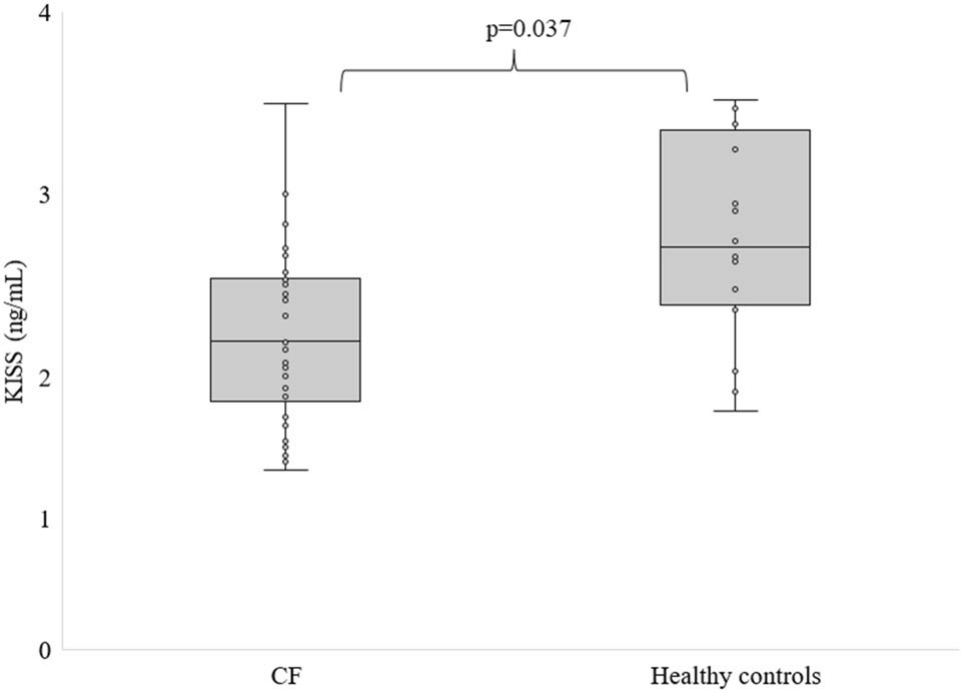
Fasting levels of KISS in CF patients and healthy individuals, differences between means were analysed using Mann–Whitney *U* test.

**Figure 2:**
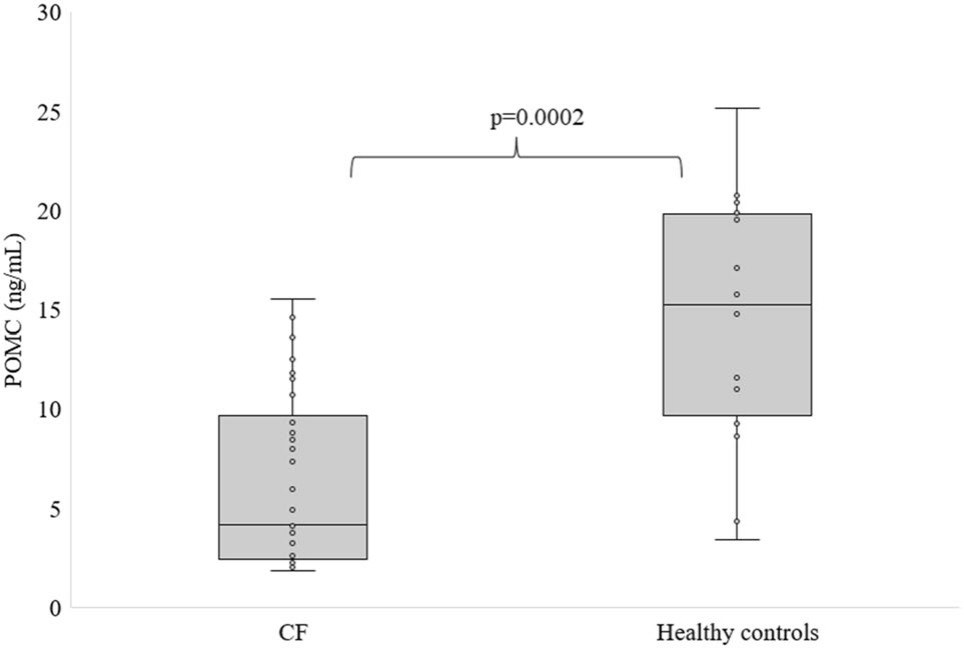
Fasting levels of POMC in CF patients and healthy individuals, differences between means were analysed using Mann–Whitney *U* test.

Figures 1 and 2 show the levels of KISS and POMC in CF patients compared to healthy subjects. Fasting KISS and POMC levels were significantly lower in patients with CF patients than in controls (1.76 ± 0.46 vs. 2.27 ± 0.56 ng/mL and 6.25 ± 4.36 vs. 14.74 ± 6.24 ng/mL).

Due to changes in hormone levels with the age of the patients, we divided patients into three age groups (below 15 years between 15 and 18 years, and above 18 years). Table 2 shows the mean hormone levels in the three age groups. There were no statistical differences in hormone levels between the study age groups, neither for KISS nor for POMC.

**Table 2:** KISS and POMC concentration depending on the age of the patients.

	< 15 years (n = 12)	15‒18 years (n = 8)	> 18 years (n = 18)
KISS (ng/mL)	1.81 ± 0.37 ^p=0.999 (A), p=0.517 (B)^	1.88 ± 0.56 ^p=0.739 (C)^	1.58 ± 0.45
POMC (ng/mL)	9.11 ± 4.7 ^p=0.197 (A), p=0.078 (B)^	4.95 ± 4.14 ^p=0.999 (C)^	4.92 ± 3.3

Data are presented as mean ± SD; differences between means were analysed using Kruskal–Wallis test.

^A^p when compared patients < 15 years and between 15 and 18 years, ^B^p when compared patients < 15 years and > 18 years, ^C^p when compared patients between 15‒18 and > 18 years.

**Figure 3:**
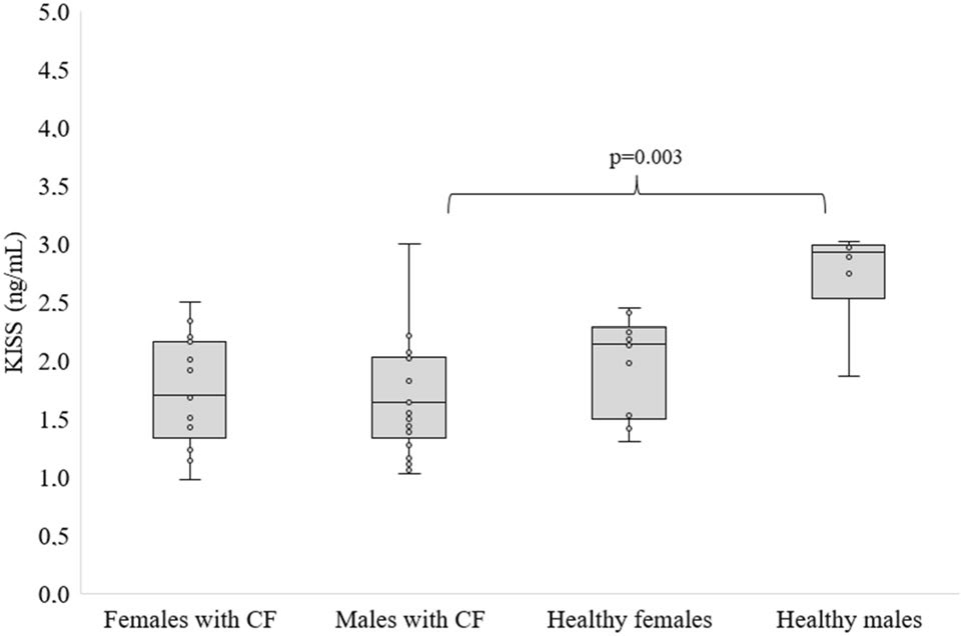
KISS levels in people with CF and healthy controls by sex, differences between means were analysed using Kruskal–Wallis test.

**Figure 4:**
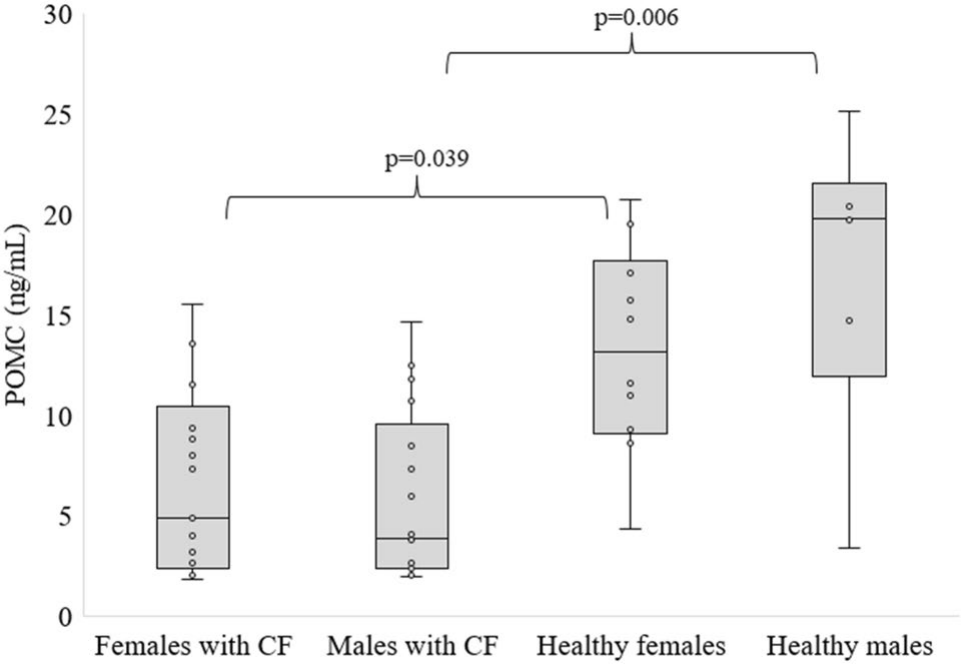
POMC levels in people with CF and healthy controls by sex, differences between means were analysed using Kruskal–Wallis test.

Figures 3 and 4 present the level of hormones in the studied groups depending on sex. Hormone levels did not differ between men and women with CF, and there was no difference in hormone levels between men and women in the healthy people. There was no difference in KISS levels between females with CF and healthy females. However, significantly decreased KISS levels were found in CF males with CF compared to males in the control group (Fig. 3).

As with KISS, POMC levels did not differ between women and men with CF, and between healthy females and males. Nevertheless, we found significantly reduced POMC levels in women and men with CF compared to men and women in the control group, respectively (Fig. 4).

Table 3 presents the correlation of KISS and POMC with the clinical parameters of the studied patients. We found a negative significant correlation of KISS and POMC with age (R = ‒ 0.3307, p < 0.05; R = − 0.355, p < 0.05) and CRP (R = ‒ 0.4536, p < 0.01; R = − 0.337, p < 0.05). Additionally, KISS was negatively correlated with CRP in patients with CF. Furthermore, we also checked whether there was a correlation between the hormones tested and leptin and neuropeptide Y, which we also determined in patients with CF. We found negative correlations between leptin and KISS, as well as leptin and POMC (R = ‒ 0.5303, p < 0.05; R = ‒ 0.5462, p < 0.001). There was no correlation between KISS levels and neuropeptide Y, as well as POMC and neuropeptide Y. KISS and POMC were not correlated.

**Table 3:** Spearman’s rank correlation coefficients and p values.

		Age	BMI	CRP	FEV_1_	Leptin	Neuropeptide Y	KISS
KISS	*R*	‒ 0.3307	‒ 0.4251	‒ 0.4536	0.3004	‒0.5303	0.204	
	p	0.0425	0.0078	0.0042	0.0668	0.012	0.2191	
POMC	*R*	‒ 0.355	‒ 0.252	‒ 0.337	0.2098	‒ 0.5462	0.2021	0.1828
	p	0.0286	0.1262	0.038	0.2061	0.0008	0.223	0.2718

*BMI* body mass index, *WBC* white blood cells, *NEU* neutrophils, *CRP* C-reactive protein, *FEV*_1_ forced expiratory volume in 1 s.

Spearman’s rank correlation coefficients and p values were estimated using Statistica software.

## Discussion

Our study describes for the first time that circulating levels of KISS and POMC in patients with CF differ from those in healthy subjects. Therefore, the main results are significantly decreased serum levels of KISS and POMC in children, adolescents and adults with CF compared to healthy subjects. Furthermore, the level of both hormones was negatively correlated with age, CRP, and serum leptin levels. KISS is involved in the regulation of puberty onset, ovarian function, trophoblast invasion, fertility regulation, pregnancy and lactation^23^. In addition to the hypothalamus, KISS and the KISS receptor are also expressed in various extrahypothalamic tissues, including the liver, pancreas, fat and gonads^24^. Consequently, the role of KISS in the reproductive process is fairly well characterized, however, it also has metabolic functions, including effects on appetite and nutritional behavior^25,26^. It is currently known that elevated levels of KISS were found in patients with pancreatic adenocarcinoma and critically ill patients during intensive care unit treatment^18,27^. We found a significantly lower level of KISS in participants with CF compared with healthy volunteers. Significantly elevated KISS levels were observed in youth with central precocious puberty^28,29^. However, puberty is delayed in people with CF and is considered one of the common clinical features of the disease, which may be related to the decreased KISS level^30^.

Furthermore, serum KISS levels were significantly higher in obese men as well as in obese/overweight girls compared to the normal weight subjects^31,32^. Maldigestion and malabsorption, especially fat and fat-soluble vitamins, poor appetite, and increased energy consumption contribute to the frequent occurrence of malnutrition in the population with CF^33^. Thus, decreased KISS levels may be associated with lower body weight in patients with CF. Animal studies have shown that KISS signaling influences body weight, energy expenditure, respiratory rate, and glucose homeostasis. Therefore, a malfunction in KISS signaling could contribute, directly or indirectly, to some aspects of human obesity, diabetes, or metabolic dysfunction^34^. A significant difference in circulating KISS levels between males and females was previously reported. An increased level of KISS in healthy females may be associated with a higher number of KISS neurons in the female hypothalamus and increased KISS production in the ovaries and adipose tissues^24^. The lack of difference in KISS levels may be due to low body fat and ovarian dysfunction in females with CF^9^. Furthermore, we found a decrease in the level of KISS in males with CF compared with healthy males. In men, circulating KISS levels change in relation to fertility status, being significantly higher in fertile men than in infertile men^35^. The primary role of hypothalamic KISS is the regulation of gonadotropin releasing hormone secretion from neurons. Gonadotropin releasing hormone acts on the gonadotrophs in the anterior pituitary gland to release follicle stimulating hormone and luteinizing hormone into the systemic circulation. The gonadotrophins then stimulate the gonads to release testosterone in males, and oestradiol as well as progesterone in females^24,36^. Hence, it seems reasonable to analyze KISS and other hormones simultaneously.

Peptides delivered from POMC play a crucial role in a variety of physiological processes, including energy homeostasis, stress response, adrenal function, sexual activity, thermoregulation, exocrine gland activity, immune system and skin pigmentation^37^. Although originally characterized as stress signal-induced neurohormones in the context of the classical hypothalamic–pituitary–adrenal stress axis, it has now been established that POMC and its derivative peptides can also be autonomously generated in many peripheral tissues^37–39^. POMC deficiency is related to early-onset severe obesity, adrenal insufficiency, skin hypopigmentation and red hair^21^. Furthermore, decreased POMC levels are also observed in peripheral nervous system tissue from diabetic patients^40^. On the other hand, an increased level of plasma POMC is characteristic of ACTH-dependent Cushing syndrome^41^. Surprisingly, patients with CF had lower serum POMC levels than the control group. Likewise, we observed significantly reduced levels of POMC in women and men with CF, indicating that sex did not influence the concentration of POMC in CF. To date, there are few reports of the determination of POMC in serum by ELISA. Interestingly, subjects with anorexia nervosa had a significantly higher level of POMC than controls (369.6 pg/mL ± 231.2 vs. 77.2 pg/mL ± 54.1, p < 0.0001)^42^.

The second goal of our work was to find the correlation between basic clinical outcomes in CF patients and the hormones tested. KISS was negatively correlated with age, BMI, CRP, and serum leptin level among participants with CF. Serum KISS levels of healthy women were found to be significantly and negatively correlated with age (R = − 0.458) in the study by Erel et al.^43^ Declined levels of KISS and its receptor in different brain regions with age were also observed in animal studies^44^. Sitticharoon et al. revealed that serum KISS was positively correlated with body weight, BMI, and serum leptin in male subjects^31^. This contrasts to our results in CF patients in whom KISS was negatively correlated with BMI and leptin. Similarly, serum KISS was negatively correlated with BMI, serum levels of insulin, glucagon, active ghrelin, and leptin in female volunteers^45^. Additionally, an inverse correlation was observed between BMI and serum KISS (R = − 0.6, p = 0.012) in the typical anorexia nervosa^46^. In patients with sepsis, KISS levels were not correlated with CRP and other serum adipokines measured, including leptin, resistin, ghrelin, or adiponectin^18^. Furthermore, KISS concentrations did not correlate with markers of systemic inflammation, including CRP in patients with pancreatic cancer^27^. It should be emphasized that the KISS level did not correlate with the spirometry results, which may indicate that the KISS level is not related to the severity of the disease. Research in cell lines has shown that KISS directly regulates neuropeptide Y synthesis and secretion via the ERK1/2 and p38 MAPK pathways, which indicates that there may be an association between the levels of the two hormones, which was not confirmed in our study^47^. In turn, in our study, POMC were negatively correlated with age and CRP in CF, but were not correlated with BMI. POMC of cerebrospinal fluid (CSF) correlated with leptin from CSF (R =  − 0.60, p < 0.001) and plasma (R =  − 0.531, p < 0.0001), insulin, BMI and adiposity in human subjects. However, CSF and plasma POMC were not correlated (R = 0.12, p = 0.52)^48^. A significant negative correlation was also detected between serum levels of POMC and leptin (R =  − 0.6631, p < 0.0001) in subjects with anorexia nervosa, similar to our study^42^. As with KISS, POMC did not correlate with spirometry results. In our study, both hormones studied did not correlate with each other or with neuropeptide Y. In conclusion, decreased KISS and POMC levels may be associated with lower body weight and delayed puberty in patients with CF.

Although to our knowledge this is the first report to describe the levels of KISS and POMC in CF, this study has certain limitations. First, the number of participants was small and not equal between the study groups. Moreover, it is a single-center study. We did not analyze other reproductive hormones. Additionally, patients with CF weighed significantly less than the healthy participants, which is also one of the limitations of the study. However, both KISS and POMC appear to be involved in the regulation of many life processes in CF, including nutrition and reproduction. Due to the longer life of the patients and the emerging diseases accompanying cystic fibrosis, it seems necessary to organize further studies to fully explore the physiology of KISS and POMC in CF.

## Data Availability

All data generated or analyzed during this study are included in this published article.
